# (*E*)-2,4,7-Trichloro-3-hydr­oxy-8-meth­oxy-1,9-dimethyl-6-(1-methyl-1-propen­yl)-11*H*-dibenzo[*b*,*e*][1,4]dioxepin-11-one monohydrate (nidulin monohydrate)

**DOI:** 10.1107/S1600536809036277

**Published:** 2009-09-16

**Authors:** Thammarat Aree, Sanya Surerum, Nattaya Ngamrojanavanich, Prasat Kittakoop

**Affiliations:** a Department of Chemistry, Faculty of Science, Chulalongkorn University, Phyathai Road, Pathumwan, Bangkok 10330, Thailand; b Chulabhorn Research Institute and Chulabhorn Graduate Institute, Vibhavadi-Rangsit Highway, Bangkok 10210, Thailand

## Abstract

In the title compound, C_20_H_17_Cl_3_O_5_·H_2_O, the nidulin mol­ecule consists of three rings, the folded central dioxepin-11-one ring being fused on both sides to phenyl rings. The mol­ecular structure is stabilized by intra­molecular O—H⋯Cl and C—H⋯Cl hydrogen bonds that generate *S*(6) ring motifs. The crystal structure is stabilized by inter­molecular O—H⋯O and O—H⋯(O,O) hydrogen bonds mediated by two inversion-related water mol­ecules, generating *R*_4_^2^(8) ring and *C*_2_^2^(4) chain motifs. Weak inter­molecular Cl⋯O halogen bonds are also present with Cl⋯O distances of 3.071 (1) and 3.182 (2) Å.

## Related literature

For the structure and synthesis of nidulin, see: Beach & Richards (1961 ▶, 1963 ▶); Bycroft & Roberts (1963 ▶). For the crystal structure of anhydrous nidulin, see: McMillan (1964 ▶). For related structures, see: Brassy *et al.* (1977 ▶); Connolly *et al.* (1984 ▶); Kawahara *et al.* (1988 ▶); Blaser & Stoeckli-Evans (1991 ▶); Xu *et al.* (2000 ▶); Lang *et al.* (2007 ▶). For the graph-set description of hydrogen-bond patterns, see: Bernstein *et al.* (1995 ▶).
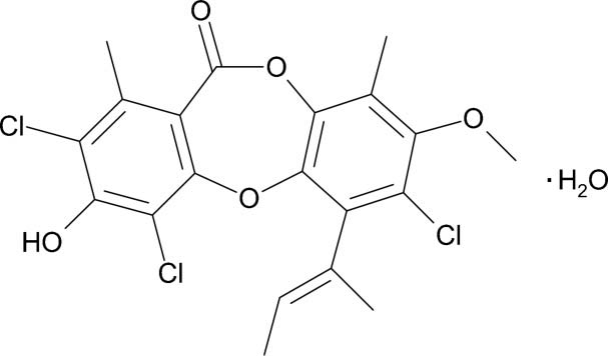


## Experimental

### 

#### Crystal data

C_20_H_17_Cl_3_O_5_·H_2_O
*M* *_r_* = 461.70
Monoclinic, 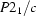

*a* = 7.7706 (4) Å
*b* = 11.0374 (5) Å
*c* = 23.9428 (10) Å
β = 96.707 (2)°
*V* = 2039.45 (16) Å^3^
*Z* = 4
Mo *K*α radiation
μ = 0.49 mm^−1^
*T* = 298 K
0.44 × 0.28 × 0.26 mm

#### Data collection

Bruker SMART APEXII CCD area-detector diffractometer
Absorption correction: multi-scan (**SADABS**; Bruker, 2005 ▶) *T*_min_ = 0.817, *T*_max_ = 0.846
11735 measured reflections
5969 independent reflections
4193 reflections with *I* > 2σ(*I*)
*R*_int_ = 0.022

#### Refinement

*R*[*F* ^2^ > 2σ(*F* ^2^)] = 0.043
*wR*(*F* ^2^) = 0.118
*S* = 1.02
5969 reflections
276 parameters
H atoms treated by a mixture of independent and constrained refinement
Δρ_max_ = 0.36 e Å^−3^
Δρ_min_ = −0.34 e Å^−3^

### 

Data collection: *APEX2* (Bruker, 2005 ▶); cell refinement: *SAINT* (Bruker, 2005 ▶); data reduction: *SAINT*; program(s) used to solve structure: *SHELXTL* (Sheldrick, 2008 ▶); program(s) used to refine structure: *SHELXTL*; molecular graphics: *ORTEP-3 for Windows* (Farrugia, 1997 ▶) and *Mercury* (Macrae *et al.* 2006 ▶); software used to prepare material for publication: *SHELXTL*.

## Supplementary Material

Crystal structure: contains datablocks I, global. DOI: 10.1107/S1600536809036277/sj2646sup1.cif

Structure factors: contains datablocks I. DOI: 10.1107/S1600536809036277/sj2646Isup2.hkl

Additional supplementary materials:  crystallographic information; 3D view; checkCIF report

## Figures and Tables

**Table 1:** Hydrogen-bond geometry (Å, °)

*D*—H⋯*A*	*D*—H	H⋯*A*	*D*⋯*A*	*D*—H⋯*A*
O1*W*—H2*W*1⋯O5	0.77 (3)	2.47 (3)	3.069 (3)	135 (3)
O1*W*—H1*W*1⋯O1^i^	0.87 (4)	2.06 (4)	2.929 (3)	177 (3)
O1*W*—H2*W*1⋯O1^ii^	0.77 (3)	2.47 (4)	3.143 (2)	147 (3)
O4—H4⋯O1*W*^iii^	0.82	1.89	2.634 (2)	150
O4—H4⋯Cl2	0.82	2.51	2.9885 (16)	119
C16—H161⋯Cl3	0.96	2.74	3.311 (3)	119

Symmetry codes: (i) 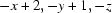; (ii) 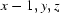; (iii) 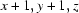.

**Fig. 1.**
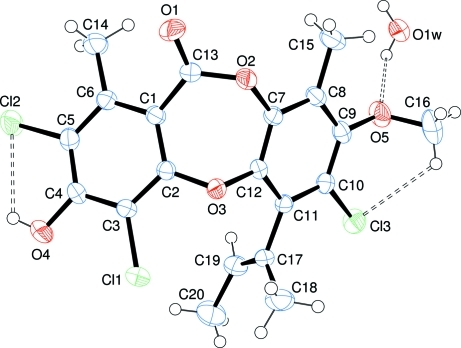
The structure of (I) with atom numbering and 50% probability displacement ellipsoids. Hydrogen bonds are shown as dashed lines.

**Fig. 2.**
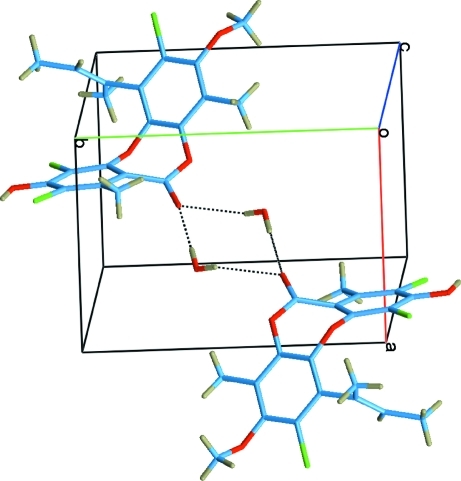
: O—H···O hydrogen bonds generating an R42(8) ring motif from inversion-related nidulin and water molecules. Hydrogen bonds are shown as dashed lines.

**Fig. 3.**
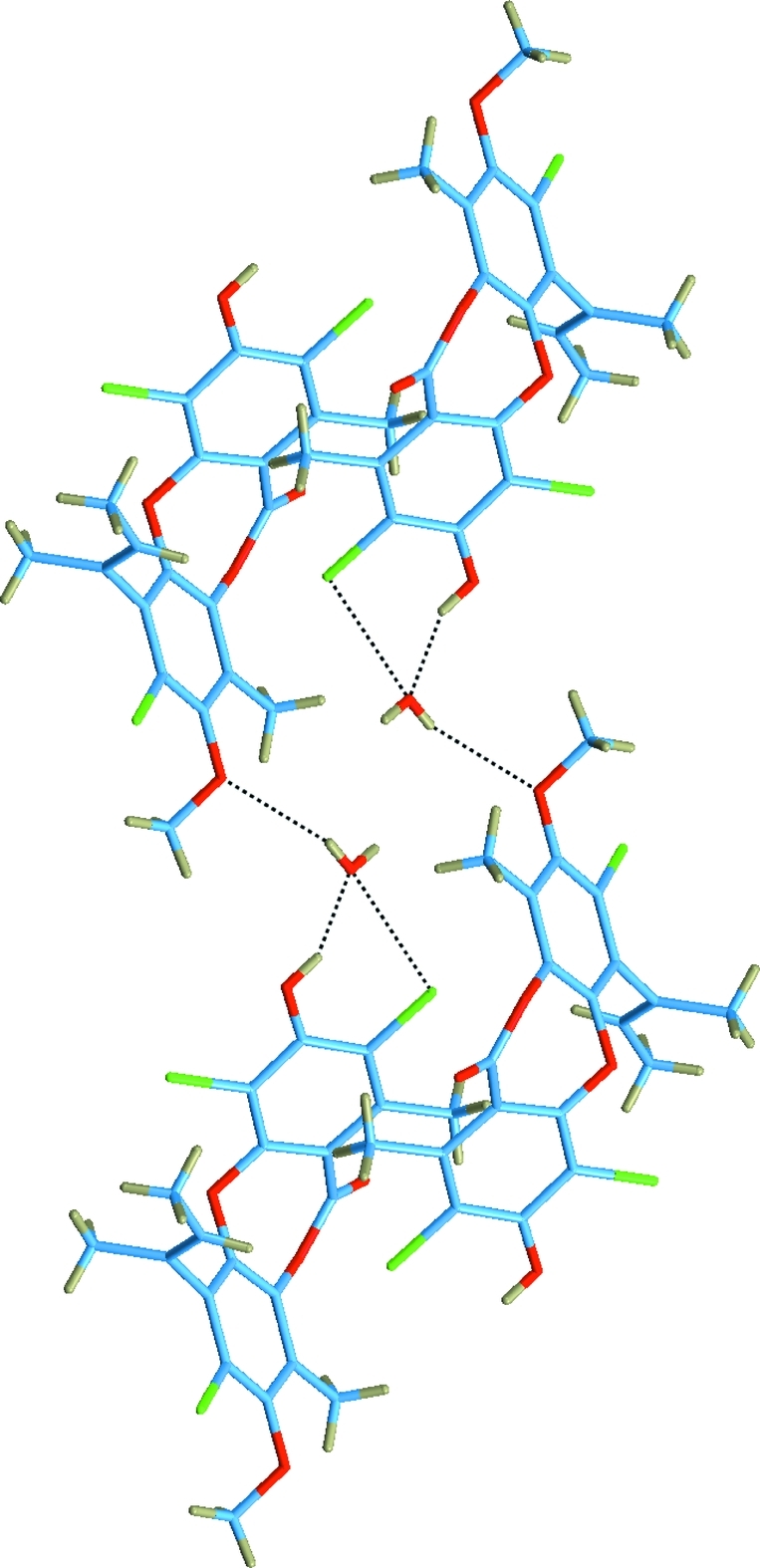
: C22(4) chains generated from the two inversion-related nidulin-monohydrate molecules through O—H···O hydrogen bonds. Intermolecular Cl···O halogen bonds are also shown. Hydrogen bonds and Cl···O interactions are shown as dashed lines.

## supplementary crystallographic information

### Comment

The title compound, (I), (*E*)-2,4,7-trichloro-3-hydroxy-8-methoxy- 1,9-dimethyl-6-(1-methyl-1-propenyl)-11*H*-dibenzo[*b*,*e*] [1,4]dioxepin-11-one (nidulin) monohydrate, C_20_H_17_Cl_3_O_5_.H_2_O (Fig. 1), is a fungal metabolite that was isolated from an identified marine sponge. The structure of the first anhydrous monoclinic crystal form of nidulin was determined and reported without atomic coordinates by McMillan (1964). Here we report a second monoclinic monohydrate crystal form; a water molecule of hydration acts as the centre of hydrogen bonding network stabilizing the entire crystal.

Because the atomic coordinates of the anhydrous form is not available, the two crystal forms of nidulin cannot be structurally compared. The molecular structures of (I) comprises three rings; the central dioxepin-11-one ring in a boat conformation is fused on both sides to the two fully substituted phenyl rings with an interplanar angle of 120.39 (7)°. The substituent atoms all lie close to the planes of the two phenyl rings. Exceptions are atoms C14, C17, Cl2 and Cl3 which deviate from the mean planes, by ≈ -0.11 Å. The methoxy C16 atom deviates from the C7···C12 mean plane by 1.229 (4) Å and the C8—C9—O5—C16 torsion angle is 105.26 (23)°. The plane of the 1-methyl-1-propenyl group defined by atoms C17, C18, C19 and C20 makes an angle of 40.23 (6)° with respect to the attached phenyl ring. For the central dioxepin-11-one ring, atoms O1, O2, O3 and C13 are displaced by 1.188 (4), 0.859 (3), 0.584 (2) and 0.669 (3) Å, respectively, from the mean plane through atoms C1, C2, C7 and C12.

The nidulin molecule is stabilized by intramolecular O4—H···Cl2 and C16—H···Cl3 hydrogen bonds each of which generates an *S*(6) ring motif. (Bernstein *et al.*, 1995) and is linked to the water molecule by an intermolecular O1W—H···O5 hydrogen bond (Fig. 1). The crystal lattice of nidulin is sustained by O—H···O intermolecular hydrogen bonds mediated by two inversion-related water molecules which generate an *R*_4_^2^(8) ring motif, (Fig. 2). *C*_2_^2^(4) chains (Bernstein *et al.*, 1995) are also formed. The structure is further stabilized by weak intermolecular Cl2···O1W and Cl3···O3 halogen bonds.

### Experimental

The title compound, (I), was extracted from the marine-derived fungus *Aspergillus sp*. CRI282–03 and single crystals of (I) were obtained from slow evaporation of an acetone-ethylacetate-hexane (1:1:1, *v*/*v*) solution at room temperature. It was found later that nidulin contained a water molecule of hydration in the crystal lattice. Water molecules found as traces in the organic solvents, are retained in the sample by strong hydrogen bonding to the nidulin molecules.

### Refinement

The water H-atoms were located in a difference electron density map and refined isotropically. All other H atoms were located and then refined using a riding model: C—H = 0.93 Å (methine), *U*_iso_(H) = 1.2*U*_eq_(C) and C—H = 0.96 Å (methyl), O—H = 0.82 Å (hydroxyl), *U*_iso_(H) = 1.5*U*_eq_(C/O).

### Crystal data

**Table:**

C_20_H_17_Cl_3_O_5_·H_2_O	*F*(000) = 952
*M**_r_* = 461.70	*D*_x_ = 1.504 Mg m^−^^3^
Monoclinic, *P*2_1_/*c*	Mo *K*α radiation, λ = 0.71073 Å
Hall symbol: -P 2ybc	Cell parameters from 4256 reflections
*a* = 7.7706 (4) Å	θ = 2.6–30.1°
*b* = 11.0374 (5) Å	µ = 0.49 mm^−^^1^
*c* = 23.9428 (10) Å	*T* = 298 K
β = 96.707 (2)°	Rod, colourless
*V* = 2039.45 (16) Å^3^	0.44 × 0.28 × 0.26 mm
*Z* = 4	

### Data collection

**Table:**

Bruker SMART APEXII CCD area-detector diffractometer	5969 independent reflections
Radiation source: fine-focus sealed tube	4193 reflections with *I* > 2σ(*I*)
graphite	*R*_int_ = 0.022
φ and ω scans	θ_max_ = 30.5°, θ_min_ = 1.7°
Absorption correction: multi-scan (*SADABS*; Bruker, 2005)	*h* = −11→8
*T*_min_ = 0.817, *T*_max_ = 0.846	*k* = −8→15
11735 measured reflections	*l* = −30→34

### Refinement

**Table:**

Refinement on *F*^2^	Primary atom site location: structure-invariant direct methods
Least-squares matrix: full	Secondary atom site location: difference Fourier map
*R*[*F*^2^ > 2σ(*F*^2^)] = 0.043	Hydrogen site location: inferred from neighbouring sites
*wR*(*F*^2^) = 0.118	H atoms treated by a mixture of independent and constrained refinement
*S* = 1.02	*w* = 1/[σ^2^(*F*_o_^2^) + (0.0513*P*)^2^ + 0.801*P*] where *P* = (*F*_o_^2^ + 2*F*_c_^2^)/3
5969 reflections	(Δ/σ)_max_ < 0.001
276 parameters	Δρ_max_ = 0.36 e Å^−^^3^
0 restraints	Δρ_min_ = −0.34 e Å^−^^3^

### Special details

**Table:**

Geometry. All e.s.d.'s (except the e.s.d. in the dihedral angle between two l.s. planes) are estimated using the full covariance matrix. The cell e.s.d.'s are taken into account individually in the estimation of e.s.d.'s in distances, angles and torsion angles; correlations between e.s.d.'s in cell parameters are only used when they are defined by crystal symmetry. An approximate (isotropic) treatment of cell e.s.d.'s is used for estimating e.s.d.'s involving l.s. planes.
Refinement. Refinement of *F*^2^ against ALL reflections. The weighted *R*-factor *wR* and goodness of fit *S* are based on *F*^2^, conventional *R*-factors *R* are based on *F*, with *F* set to zero for negative *F*^2^. The threshold expression of *F*^2^ > σ(*F*^2^) is used only for calculating *R*-factors(gt) *etc*. and is not relevant to the choice of reflections for refinement. *R*-factors based on *F*^2^ are statistically about twice as large as those based on *F*, and *R*- factors based on ALL data will be even larger.

### Fractional atomic coordinates and isotropic or equivalent isotropic displacement parameters (Å^2^)

**Table:**

	*x*	*y*	*z*	*U*_iso_*/*U*_eq_	
Cl1	1.19555 (8)	1.09399 (5)	0.19154 (2)	0.04719 (16)	
Cl2	1.27489 (9)	1.13429 (5)	−0.02688 (2)	0.04800 (16)	
Cl3	0.51380 (6)	0.74321 (5)	0.19233 (2)	0.04035 (13)	
C1	1.2062 (2)	0.84575 (16)	0.07006 (7)	0.0260 (3)	
C2	1.1823 (2)	0.90311 (15)	0.12065 (7)	0.0242 (3)	
C3	1.2058 (2)	1.02676 (17)	0.12732 (8)	0.0294 (4)	
C4	1.2405 (2)	1.09852 (17)	0.08192 (8)	0.0318 (4)	
C5	1.2461 (2)	1.04181 (17)	0.02995 (8)	0.0312 (4)	
C6	1.2317 (2)	0.91718 (16)	0.02277 (8)	0.0279 (4)	
C7	1.0090 (2)	0.66126 (15)	0.12272 (7)	0.0254 (3)	
C8	0.8747 (2)	0.57758 (16)	0.11380 (8)	0.0292 (4)	
C9	0.7239 (2)	0.60330 (17)	0.13765 (8)	0.0298 (4)	
C10	0.7088 (2)	0.71040 (16)	0.16780 (7)	0.0269 (4)	
C11	0.8453 (2)	0.79319 (15)	0.17737 (7)	0.0234 (3)	
C12	0.9971 (2)	0.76342 (15)	0.15515 (7)	0.0238 (3)	
C13	1.2414 (2)	0.71340 (17)	0.06833 (8)	0.0291 (4)	
C14	1.2365 (3)	0.8636 (2)	−0.03500 (8)	0.0418 (5)	
H143	1.1706	0.9138	−0.0624	0.063*	
H141	1.1876	0.7837	−0.0361	0.063*	
H142	1.3543	0.8594	−0.0432	0.063*	
C15	0.8942 (3)	0.46381 (19)	0.08076 (10)	0.0446 (5)	
H152	0.8695	0.4809	0.0413	0.067*	
H151	0.8147	0.4036	0.0913	0.067*	
H153	1.0106	0.4342	0.0886	0.067*	
C16	0.5569 (4)	0.4478 (2)	0.17417 (12)	0.0629 (7)	
H162	0.6588	0.4005	0.1852	0.094*	
H163	0.4611	0.3948	0.1630	0.094*	
H161	0.5310	0.4967	0.2053	0.094*	
C17	0.8290 (2)	0.91073 (16)	0.20689 (8)	0.0282 (4)	
C18	0.9100 (4)	0.9166 (2)	0.26682 (9)	0.0496 (6)	
H181	0.9067	0.9985	0.2801	0.074*	
H183	1.0282	0.8899	0.2691	0.074*	
H182	0.8471	0.8652	0.2896	0.074*	
C19	0.7478 (3)	1.00046 (18)	0.17871 (9)	0.0404 (5)	
H19	0.7012	0.9828	0.1421	0.061*	
C20	0.7211 (4)	1.1273 (2)	0.19822 (13)	0.0623 (7)	
H201	0.7663	1.1347	0.2371	0.093*	
H202	0.5995	1.1457	0.1938	0.093*	
H203	0.7803	1.1829	0.1762	0.093*	
O1	1.3465 (2)	0.67026 (14)	0.04114 (6)	0.0449 (4)	
O2	1.16197 (17)	0.63383 (11)	0.10058 (6)	0.0321 (3)	
O3	1.14180 (15)	0.83871 (11)	0.16655 (5)	0.0265 (3)	
O4	1.2591 (3)	1.21752 (13)	0.09119 (7)	0.0519 (4)	
H4	1.2950	1.2497	0.0639	0.078*	
O5	0.58658 (19)	0.52480 (13)	0.12786 (6)	0.0418 (4)	
O1W	0.3826 (3)	0.38791 (17)	0.02997 (9)	0.0617 (6)	
H1W1	0.465 (5)	0.372 (3)	0.0093 (15)	0.089 (12)*	
H2W1	0.396 (4)	0.449 (3)	0.0451 (14)	0.076 (11)*	

### Atomic displacement parameters (Å^2^)

**Table:**

	*U*^11^	*U*^22^	*U*^33^	*U*^12^	*U*^13^	*U*^23^
Cl1	0.0688 (4)	0.0363 (3)	0.0391 (3)	−0.0159 (2)	0.0170 (3)	−0.0148 (2)
Cl2	0.0726 (4)	0.0353 (3)	0.0363 (3)	−0.0106 (3)	0.0073 (3)	0.0068 (2)
Cl3	0.0276 (2)	0.0440 (3)	0.0510 (3)	−0.0006 (2)	0.0115 (2)	0.0000 (2)
C1	0.0256 (8)	0.0231 (8)	0.0295 (9)	−0.0007 (6)	0.0044 (7)	−0.0032 (7)
C2	0.0215 (8)	0.0244 (8)	0.0268 (8)	−0.0018 (6)	0.0036 (6)	−0.0019 (6)
C3	0.0320 (9)	0.0270 (9)	0.0300 (9)	−0.0050 (7)	0.0075 (7)	−0.0073 (7)
C4	0.0348 (10)	0.0239 (9)	0.0374 (10)	−0.0070 (7)	0.0067 (8)	−0.0026 (7)
C5	0.0324 (10)	0.0303 (9)	0.0307 (9)	−0.0045 (7)	0.0032 (8)	0.0034 (7)
C6	0.0287 (9)	0.0279 (9)	0.0272 (9)	−0.0019 (7)	0.0029 (7)	−0.0027 (7)
C7	0.0272 (8)	0.0207 (8)	0.0288 (9)	0.0032 (6)	0.0057 (7)	0.0012 (6)
C8	0.0366 (10)	0.0209 (8)	0.0293 (9)	−0.0009 (7)	0.0008 (7)	−0.0009 (7)
C9	0.0305 (9)	0.0259 (9)	0.0318 (9)	−0.0064 (7)	−0.0017 (7)	0.0018 (7)
C10	0.0241 (8)	0.0279 (9)	0.0288 (9)	−0.0004 (7)	0.0038 (7)	0.0031 (7)
C11	0.0253 (8)	0.0226 (8)	0.0225 (8)	0.0009 (6)	0.0039 (6)	0.0010 (6)
C12	0.0244 (8)	0.0210 (8)	0.0259 (8)	−0.0024 (6)	0.0026 (6)	0.0014 (6)
C13	0.0320 (9)	0.0258 (9)	0.0304 (9)	0.0013 (7)	0.0079 (7)	−0.0029 (7)
C14	0.0591 (14)	0.0368 (11)	0.0298 (10)	−0.0036 (10)	0.0063 (9)	−0.0035 (8)
C15	0.0521 (13)	0.0299 (11)	0.0529 (13)	−0.0047 (9)	0.0107 (11)	−0.0136 (9)
C16	0.0649 (17)	0.0448 (14)	0.0782 (19)	−0.0249 (12)	0.0053 (14)	0.0144 (13)
C17	0.0316 (9)	0.0261 (9)	0.0281 (9)	−0.0019 (7)	0.0095 (7)	−0.0044 (7)
C18	0.0693 (16)	0.0460 (13)	0.0322 (11)	−0.0012 (11)	0.0007 (11)	−0.0094 (9)
C19	0.0502 (12)	0.0298 (10)	0.0430 (12)	0.0058 (9)	0.0126 (10)	−0.0050 (8)
C20	0.0777 (19)	0.0327 (12)	0.0809 (19)	0.0149 (12)	0.0276 (16)	−0.0084 (12)
O1	0.0545 (9)	0.0367 (8)	0.0484 (9)	0.0107 (7)	0.0267 (8)	−0.0013 (7)
O2	0.0348 (7)	0.0214 (6)	0.0423 (8)	0.0042 (5)	0.0132 (6)	−0.0001 (5)
O3	0.0264 (6)	0.0274 (6)	0.0260 (6)	−0.0053 (5)	0.0044 (5)	−0.0020 (5)
O4	0.0855 (13)	0.0249 (7)	0.0483 (9)	−0.0148 (8)	0.0211 (9)	−0.0054 (6)
O5	0.0374 (8)	0.0385 (8)	0.0482 (9)	−0.0166 (6)	−0.0003 (7)	−0.0030 (7)
O1W	0.0887 (15)	0.0334 (9)	0.0700 (13)	−0.0188 (9)	0.0389 (12)	−0.0100 (9)

### Geometric parameters (Å, °)

**Table:**

Cl1—C3	1.7175 (18)	C13—O2	1.364 (2)
Cl2—C5	1.7361 (19)	C14—H143	0.9600
Cl3—C10	1.7261 (18)	C14—H141	0.9600
C1—C2	1.398 (2)	C14—H142	0.9600
C1—C6	1.412 (2)	C15—H152	0.9600
C1—C13	1.488 (2)	C15—H151	0.9600
C2—O3	1.376 (2)	C15—H153	0.9600
C2—C3	1.384 (2)	C16—O5	1.437 (3)
C3—C4	1.397 (3)	C16—H162	0.9600
C4—O4	1.337 (2)	C16—H163	0.9600
C4—C5	1.398 (3)	C16—H161	0.9600
C5—C6	1.389 (3)	C17—C19	1.317 (3)
C6—C14	1.509 (3)	C17—C18	1.499 (3)
C7—C12	1.378 (2)	C18—H181	0.9600
C7—O2	1.390 (2)	C18—H183	0.9600
C7—C8	1.391 (3)	C18—H182	0.9600
C8—C9	1.391 (3)	C19—C20	1.498 (3)
C8—C15	1.501 (3)	C19—H19	0.9300
C9—O5	1.373 (2)	C20—H201	0.9600
C9—C10	1.397 (3)	C20—H202	0.9600
C10—C11	1.399 (2)	C20—H203	0.9600
C11—C12	1.389 (2)	O4—H4	0.8200
C11—C17	1.490 (2)	O1W—H1W1	0.87 (4)
C12—O3	1.399 (2)	O1W—H2W1	0.77 (3)
C13—O1	1.201 (2)		
			
C2—C1—C6	119.13 (16)	C6—C14—H141	109.5
C2—C1—C13	120.81 (16)	H143—C14—H141	109.5
C6—C1—C13	118.85 (16)	C6—C14—H142	109.5
O3—C2—C3	117.18 (15)	H143—C14—H142	109.5
O3—C2—C1	121.60 (15)	H141—C14—H142	109.5
C3—C2—C1	121.16 (16)	C8—C15—H152	109.5
C2—C3—C4	120.29 (16)	C8—C15—H151	109.5
C2—C3—Cl1	120.68 (14)	H152—C15—H151	109.5
C4—C3—Cl1	119.03 (14)	C8—C15—H153	109.5
O4—C4—C3	117.05 (17)	H152—C15—H153	109.5
O4—C4—C5	125.00 (17)	H151—C15—H153	109.5
C3—C4—C5	117.89 (16)	O5—C16—H162	109.5
C6—C5—C4	122.89 (17)	O5—C16—H163	109.5
C6—C5—Cl2	120.08 (15)	H162—C16—H163	109.5
C4—C5—Cl2	117.03 (14)	O5—C16—H161	109.5
C5—C6—C1	118.06 (16)	H162—C16—H161	109.5
C5—C6—C14	119.41 (17)	H163—C16—H161	109.5
C1—C6—C14	122.48 (16)	C19—C17—C11	118.29 (17)
C12—C7—O2	120.62 (15)	C19—C17—C18	125.47 (19)
C12—C7—C8	122.10 (16)	C11—C17—C18	116.24 (17)
O2—C7—C8	117.15 (15)	C17—C18—H181	109.5
C7—C8—C9	117.01 (16)	C17—C18—H183	109.5
C7—C8—C15	121.09 (18)	H181—C18—H183	109.5
C9—C8—C15	121.90 (17)	C17—C18—H182	109.5
O5—C9—C8	118.43 (17)	H181—C18—H182	109.5
O5—C9—C10	120.75 (17)	H183—C18—H182	109.5
C8—C9—C10	120.69 (16)	C17—C19—C20	128.2 (2)
C9—C10—C11	121.96 (16)	C17—C19—H19	115.9
C9—C10—Cl3	118.92 (14)	C20—C19—H19	115.9
C11—C10—Cl3	119.10 (14)	C19—C20—H201	109.5
C12—C11—C10	116.40 (15)	C19—C20—H202	109.5
C12—C11—C17	120.72 (15)	H201—C20—H202	109.5
C10—C11—C17	122.79 (15)	C19—C20—H203	109.5
C7—C12—C11	121.66 (16)	H201—C20—H203	109.5
C7—C12—O3	119.40 (15)	H202—C20—H203	109.5
C11—C12—O3	118.93 (15)	C13—O2—C7	122.63 (14)
O1—C13—O2	115.66 (17)	C2—O3—C12	113.85 (13)
O1—C13—C1	122.90 (17)	C4—O4—H4	109.5
O2—C13—C1	121.33 (15)	C9—O5—C16	115.65 (17)
C6—C14—H143	109.5	H1W1—O1W—H2W1	112 (3)
			
C6—C1—C2—O3	174.18 (15)	O5—C9—C10—Cl3	−0.4 (2)
C13—C1—C2—O3	−18.5 (3)	C8—C9—C10—Cl3	175.49 (14)
C6—C1—C2—C3	−8.7 (3)	C9—C10—C11—C12	0.1 (3)
C13—C1—C2—C3	158.65 (17)	Cl3—C10—C11—C12	−178.12 (13)
O3—C2—C3—C4	−177.53 (16)	C9—C10—C11—C17	176.83 (17)
C1—C2—C3—C4	5.2 (3)	Cl3—C10—C11—C17	−1.4 (2)
O3—C2—C3—Cl1	3.3 (2)	O2—C7—C12—C11	179.51 (15)
C1—C2—C3—Cl1	−173.97 (14)	C8—C7—C12—C11	−4.8 (3)
C2—C3—C4—O4	178.96 (18)	O2—C7—C12—O3	−1.1 (2)
Cl1—C3—C4—O4	−1.9 (3)	C8—C7—C12—O3	174.52 (16)
C2—C3—C4—C5	1.6 (3)	C10—C11—C12—C7	3.6 (3)
Cl1—C3—C4—C5	−179.24 (15)	C17—C11—C12—C7	−173.18 (16)
O4—C4—C5—C6	177.9 (2)	C10—C11—C12—O3	−175.78 (15)
C3—C4—C5—C6	−5.0 (3)	C17—C11—C12—O3	7.4 (2)
O4—C4—C5—Cl2	−1.4 (3)	C2—C1—C13—O1	−138.3 (2)
C3—C4—C5—Cl2	175.76 (15)	C6—C1—C13—O1	29.0 (3)
C4—C5—C6—C1	1.5 (3)	C2—C1—C13—O2	37.8 (3)
Cl2—C5—C6—C1	−179.23 (14)	C6—C1—C13—O2	−154.90 (17)
C4—C5—C6—C14	179.14 (19)	C12—C11—C17—C19	98.9 (2)
Cl2—C5—C6—C14	−1.6 (3)	C10—C11—C17—C19	−77.7 (2)
C2—C1—C6—C5	5.2 (3)	C12—C11—C17—C18	−80.3 (2)
C13—C1—C6—C5	−162.32 (17)	C10—C11—C17—C18	103.1 (2)
C2—C1—C6—C14	−172.29 (18)	C11—C17—C19—C20	−177.4 (2)
C13—C1—C6—C14	20.1 (3)	C18—C17—C19—C20	1.7 (4)
C12—C7—C8—C9	2.1 (3)	O1—C13—O2—C7	−162.93 (18)
O2—C7—C8—C9	177.87 (16)	C1—C13—O2—C7	20.7 (3)
C12—C7—C8—C15	−176.85 (18)	C12—C7—O2—C13	−54.8 (2)
O2—C7—C8—C15	−1.0 (3)	C8—C7—O2—C13	129.38 (18)
C7—C8—C9—O5	177.59 (16)	C3—C2—O3—C12	129.37 (17)
C15—C8—C9—O5	−3.5 (3)	C1—C2—O3—C12	−53.4 (2)
C7—C8—C9—C10	1.6 (3)	C7—C12—O3—C2	70.77 (19)
C15—C8—C9—C10	−179.45 (19)	C11—C12—O3—C2	−109.84 (17)
O5—C9—C10—C11	−178.63 (16)	C8—C9—O5—C16	105.3 (2)
C8—C9—C10—C11	−2.8 (3)	C10—C9—O5—C16	−78.8 (3)

### Hydrogen-bond geometry (Å, °)

**Table:**

*D*—H···*A*	*D*—H	H···*A*	*D*···*A*	*D*—H···*A*
O1W—H2W1···O5	0.77 (3)	2.47 (3)	3.069 (3)	135 (3)
O1W—H1W1···O1^i^	0.87 (4)	2.06 (4)	2.929 (3)	177 (3)
O1W—H2W1···O1^ii^	0.77 (3)	2.47 (4)	3.143 (2)	147 (3)
O4—H4···O1W^iii^	0.82	1.89	2.634 (2)	150
O4—H4···Cl2	0.82	2.51	2.9885 (16)	119
C16—H161···Cl3	0.96	2.74	3.311 (3)	119

Symmetry codes: (i) −*x*+2, −*y*+1, −*z*; (ii) *x*−1, *y*, *z*; (iii) *x*+1, *y*+1, *z*.

## References

[bb1] Beach, W. F. & Richards, J. H. (1961). *J. Org. Chem.* **26**, 1339–1340.

[bb2] Beach, W. F. & Richards, J. H. (1963). *J. Org. Chem.* **28**, 2746–2751.

[bb3] Bernstein, J., Davis, R. E., Shimoni, L. & Chang, N.-L. (1995). *Angew. Chem. Int. Ed. Engl.* **34**, 1555–1573.

[bb4] Blaser, D. & Stoeckli-Evans, H. (1991). *Acta Cryst.* C**47**, 2624–2626.

[bb6] Brassy, C., Bodo, B. & Molho, D. (1977). *Acta Cryst.* B**33**, 2559–2562.

[bb7] Bruker (2005). *APEX2*, *SAINT* and *SADABS* Bruker AXS Inc., Madison, Wisconsin, USA.

[bb8] Bycroft, B. W. & Roberts, J. C. (1963). *J. Org. Chem.* **28**, 1429–1430.

[bb9] Connolly, J. D., Freer, A. A., Kalb, K. & Huneck, S. (1984). *Phytochemistry*, **23**, 857–858.

[bb10] Farrugia, L. J. (1997). *J. Appl. Cryst.* **30**, 565.

[bb11] Kawahara, N., Nakajima, S., Satoh, Y., Yamazaki, M. & Kawai, K.-I. (1988). *Chem. Pharm. Bull.* **36**, 1970–1975.

[bb12] Lang, G., Cole, A. L. J., Blunt, J. W., Robinson, W. T. & Munro, M. H. G. (2007). *J. Nat. Prod.* **70**, 310–311. 10.1021/np060202u 17315967

[bb13] Macrae, C. F., Edgington, P. R., McCabe, P., Pidcock, E., Shields, G. P., Taylor, R., Towler, M. & van de Streek, J. (2006). *J. Appl. Cryst.* **39**, 453–457.

[bb14] McMillan, J. A. S. (1964). *Diss. Abstr.* **25**, 868. Order No. 64-8413, 98 pp. Univ. Microfilms, Ann Arbor, Michigan, USA.

[bb15] Sheldrick, G. M. (2008). *Acta Cryst.* A**64**, 112–122. 10.1107/S0108767307043930 18156677

[bb16] Xu, Y. J., Chiang, P. Y., Lai, Y. H., Vittal, J. J., Wu, X. H., Tan, B. K. H., Imiyabir, Z. & Goh, S. H. (2000). *J. Nat. Prod.* **63**, 1361–1363. 10.1021/np000141e 11076552

